# Mental health literacy as a moderator: association between psychological vulnerability and adolescent anxiety

**DOI:** 10.3389/fpsyg.2025.1521224

**Published:** 2025-06-03

**Authors:** Qianying Hu, Yingyan Zhong, Jianhua Chen, Rumeng Chen, Enzhao Cong, Yifeng Xu

**Affiliations:** ^1^Shanghai Mental Health Center, Shanghai Jiao Tong University School of Medicine, Shanghai, China; ^2^Shanghai Tenth People's Hospital, School of Medicine, Tongji University, Shanghai, China

**Keywords:** psychological vulnerability, anxiety, mental health literacy, adolescent, moderator

## Abstract

**Background:**

Adolescent anxiety’s underlying mechanisms remain unclear, which undermines adolescents’ social functioning. This study examined the moderating role of mental health literacy in the relationship between adolescent anxiety and psychological vulnerability.

**Methods:**

A cross-sectional study was conducted on 1,591 middle and high school students through online questionnaires in January 2023 in Guizhou Province, China. The Psychological Vulnerability Scale, Mental Health Literacy Scale, and Screen for Child Anxiety-Related Emotional Disorders were used to measure psychological vulnerability, mental health literacy, anxiety, and its five dimensions, including panic/somatic, generalized anxiety, separation anxiety, social phobia, and school phobia in adolescents, respectively.

**Results:**

Psychological vulnerability was significantly correlated with adolescent anxiety and its dimensions (*p* < 0.05). The moderating role of mental health literacy on the relationship between psychological vulnerability and adolescent anxiety was left marginally significant (*p* = 0.07). Furthermore, mental health literacy significantly weakened the relationship between psychological vulnerability and social phobia (*p* < 0.05). The upward trend of anxiety as psychological vulnerability increases among the high mental health literacy group was significantly slower than the low mental health literacy group.

**Conclusion:**

Those findings suggest educators should develop curriculum resources to strengthen the cultivation of mental health literacy among adolescents to promote adolescent mental health.

## 1 Introduction

Adolescent anxiety is an internalizing disorder characterized by excessive worry and fear, with a prevalence of approximately 15–20% (Doering et al., 2022). Its prevalence has been rising (Racine et al., 2021; Biswas et al., 2020), even higher than adult anxiety (Kalin, 2020). Notably, anxiety rates among Chinese adolescents surged during the COVID-19 pandemic, ranging from 19 to 36.7% (Chen et al., 2021). The years lived with disability due to adolescent anxiety have increased to 12.46, reflecting a substantial disease burden (Klaufus et al., 2022). Adolescents with anxiety symptoms often face additional adaptive challenges, such as sleep problems (Crowe and Spiro-Levitt, 2021), Internet addiction (Kim et al., 2018), and substance abuse (Wood et al., 2020).

Psychological vulnerability is a “pattern of cognitive beliefs reflecting a dependence on achievement or external sources of affirmation for one’s sense of self-worth” (Sinclair and Wallston, 1999). It refers to the susceptibility to negative outcomes and stress within cognitive structures (Wright et al., 2012). Characterized by risk perception and emotional safety, psychological vulnerability varies among individuals, but excessively high levels can increase the likelihood of psychological issues under stress (Zubin and Spring, 1977; Wright et al., 2012). Previous research has shown a significant association between adolescent anxiety symptoms and high psychological vulnerability, often shaped by early environmental factors (Chorpita and Barlow, 2018; Gallagher et al., 2014).

Although anxiety poses a serious threat to the mental health of adolescents, it is challenging for them to identify their own or others’ anxiety symptoms (Paulus et al., 2015; Coles et al., 2016), with low recognition rates (Hart et al., 2022; Yu et al., 2016). Moreover, adolescents with anxiety disorder often fail to receive any form of professional assistance (Alonso et al., 2007; Zhao and Hu, 2022). Their help-seeking behavior was very limited (Heinig et al., 2021), possibly due to the heightened stigma associated with their condition (Hart et al., 2022; Lynch et al., 2021). The recognition and help-seeking by adolescents for anxiety disorders precisely exemplify an individual’s mental health literacy (MHL), which is defined as knowledge about mental health disorders associated with their recognition, management, and prevention (Jorm et al., 1997). Nowadays, people are more knowledgeable about physical than mental health (Wickstead and Furnham, 2017). There is a gap between the general public’s perceptions of mental health and illness and established academic knowledge, which can affect when and how the public seeks help for mental health issues (Hughner and Kleine, 2004). Lower mental health literacy is associated with more severe anxiety symptoms (Huang et al., 2021; Ding et al., 2022). Furthermore, higher levels of mental health literacy are likely to enhance adolescents’ recognition and understanding of anxiety symptoms (Furnham and Lousley, 2013; Morgado et al., 2021), which is beneficial to seeking help at the appropriate times (Coles and Coleman, 2010). In addition, previous research showed that the development of mental health literacy in adolescents can reduce certain modifiable psychological vulnerabilities, thereby promoting the improvement of adolescents’ mental health (Nogueira et al., 2023; Teixeira et al., 2022).

To sum up, the mechanisms underlying the correlation between anxiety and psychological vulnerability have not been completely elucidated, specifically the moderating effects of certain variables on this relationship. Hence, mental health literacy might have the potential to attenuate the connection between psychological vulnerability and adolescent anxiety. This study hypothesizes that (1) Psychological vulnerability is positively associated with adolescent anxiety; (2) Mental health literacy played a significant moderating role in the relationship between psychological vulnerability and adolescent anxiety; (3) For adolescents with high mental health literacy, as psychological vulnerability increased, their anxiety levels showed a significant lower upward trend than adolescents with low mental health literacy.

## 2 Materials and methods

### 2.1 Participants and procedure

This cross-sectional study was conducted in January 2023 among middle and high school students in Guizhou Province, China. The research was carried out online, and all questionnaires were created, distributed, and collected using the online survey tool, Wenjuanxing. The sample size was calculated based on a 25% prevalence rate of anxiety symptoms in Chinese adolescents since the outbreak of the COVID-19 pandemic in 2021 (Chai et al., 2021) and a minimum required sample size of 298 for epidemiological surveys (Zhao and Hu, 2022). The minimum required sample size was determined to be 1,192 participants. Convenience sampling was used, and the questionnaires for online surveys were distributed by school principals through WeChat. The informed consent form was placed at the beginning of the questionnaire, and consent from legal guardians was obtained. Participants are anonymous and voluntary for the questionnaire.

### 2.2 Research instruments

#### 2.2.1 Socio-demographic variables questionnaire

A self-report questionnaire was used to collect individual-level variables, including gender and age. Family-level variables included having siblings, the family’s economic status, parents’ marital status, and living area. Gender was categorized as male or female, and age was divided into five groups: <=12, 13, 14, 15, > = 16. The family’s economic status was divided into five categories: very poor, poor, moderate, wealthy, and very wealthy. Parents’ marital status was categorized as original marriage, remarried, divorced/separated, and widowed. The living area was classified as rural or urban.

#### 2.2.2 Psychological vulnerability

The psychological vulnerability was measured using the Psychological Vulnerability Scale (PVS) developed by Nogueira, Barros, and Sequeira (Nogueira et al., 2017). The scale consisted of six items that participants rated on a 5-point scale, with higher scores indicating greater psychological vulnerability. In this study, the PVS showed good internal consistency with a Cronbach’s *α* of 0.84.

#### 2.2.3 Mental health literacy

Mental health literacy was measured using the Mental Health Literacy Scale developed by O’Connor and Casey, based on Jorm’s concept of MHL (O'Connor and Casey, 2015). The original scale consisted of 35 items, but the Chinese version, adapted by Qiu and Liu, included 23 items (Qiu and Liu, 2021). The scale comprised four dimensions. The first dimension was rated on a 4-point scale, while the other three dimensions used a 5-point scale. In this study, Cronbach’s *α* for MHLS was 0.78.

#### 2.2.4 Anxiety

Anxiety was assessed using the Screen for Child Anxiety-Related Emotional Disorders (SCARED)(Birmaher et al., 1997), suitable for children and adolescents aged 8 to 16 years. It comprised 41 items rated on a 3-point scale (0 for “no symptoms” and 2 for “often present”). The total score ranged from 0 to 82, with higher scores indicating more severe anxiety symptoms. The SCARED also had five dimensions: panic/somatic, generalized anxiety, separation anxiety, social phobia, and school phobia. The cutoff score for anxiety was 23. The Chinese version of SCARED has been validated for good reliability and validity in Chinese children and adolescents (Wang et al., 2002), and in this study, Cronbach’s α for SCARED was 0.95.

### 2.3 Quality control questions

To ensure the quality of the study, three quality control questions were included in the questionnaire. These questions included a two-digit arithmetic calculation, two designated response options, and were placed at the 25, 50, and 75% marks of the questionnaire length. Only questionnaires with correct answers to all quality control questions were considered valid, and those with response times of less than 2 s per question were excluded (Huang et al., 2012). The Common Method Bias Test via a Harman single-factor test was performed. The results indicated that the unrotated first factor explained 21.04% of the variance, which was lower than the critical standard of 40%, suggesting no significant common method bias in this study.

### 2.4 Data analysis

All statistical analyses were conducted using SPSS software version 24.0 (Chicago, IL, USA). Continuous variables were presented as means and standard deviations (SD). Due to the non-normal distribution of anxiety scores, non-parametric tests such as the Mann–Whitney U test or Kruskal-Wallis one-way ANOVA were used to compare anxiety and its dimensions scores among socio-demographic variables. The chi-square test was used to compare the prevalence of anxiety symptoms among different demographic characteristics. Bonferroni correction was applied for *post hoc* pairwise comparisons. Spearman correlations were used to analyze the relationships between psychological vulnerability, mental health literacy, covariates, and anxiety and its dimensions. The moderating effect of mental health literacy was analyzed using Model 1 of PROCESS 3.4. A *p*-value less than 0.05 was considered statistically significant.

## 3 Results

### 3.1 Distribution of anxiety in adolescents’ socio-demographic characteristics

A total of 2,303 questionnaires were collected, and after applying the quality control criteria, 1,591 valid questionnaires were retained (Table 1). Among the respondents, 876 were male (55.06%), and 715 were female (44.94%). In terms of age, 116 respondents (7.29%) were under 12 years old, 365 (22.94%) were 13 years old, 447 (28.10%) were 14 years old, 432 (27.15%) were 15 years old, and 231 (14.52%) were above 16 years old. The majority of respondents had siblings (*N* = 1,494, 93.90%), and most of their parents were in original marriages (*N* = 1,312, 82.46%). A small percentage of respondents’ parents were remarried (*N* = 103, 6.47%), divorced/separated (*N* = 159, 9.55%), or widowed (*N* = 24, 1.51%). About one-third of respondents reported poor family economic status (*N* = 348, 21.87%) or even very poor (*N* = 130, 8.17%). Only a few respondents reported being in wealthy (*N* = 41, 2.58%) or very wealthy (*N* = 3, 0.19%) families. More than half of the respondents lived in rural areas (*N* = 862, 54.18%), while 729 (45.82%) lived in urban areas. Age was the only variable showing significant differences in anxiety scores (*p* = 0.03) and social phobia scores (*p* = 0.04), while the other socio-demographic characteristics that influenced anxiety did not significantly differ.

**Table 1:** Comparison of anxiety based on socio-demographic characteristics among adolescents (*N* = 1,591).

Variables	*n* (%)	The score of anxiety		Panic/somatic		Generalized anxiety		Separation anxiety		Social phobia		School phobia	
		M (SD)	*p*	*M* (SD)	*p*	*M* (SD)	*p*	*M* (SD)	*p*	*M* (SD)	*p*	*M* (SD)	*p*
Gender													
Male	876 (55.1%)	16.20 ± 13.96	0.37	4.04 ± 4.26	0.20	3.87 ± 4.13	0.44	2.24 ± 2.61	0.62	4.83 ± 4.00	0.35	1.22 ± 1.66	0.64
Female	715 (44.9%)	16.93 ± 14.35		4.30 ± 4.35		4.07 ± 4.32		2.35 ± 2.73		5.00 ± 3.98		1.21 ± 1.67	
Age (years)													
<=12	116 (7.3%)	16.70 ± 15.39	0.03*	4.06 ± 4.43	0.36	4.13 ± 4.52	0.11	2.20 ± 2.61	0.16	4.97 ± 4.34	0.04*	1.34 ± 1.85	0.06
13	365 (22.9%)	16.39 ± 14.15		4.47 ± 4.28		3.92 ± 4.31		2.21 ± 2.48		4.92 ± 4.09		1.17 ± 1.63	
14	447 (28.1%)	16.29 ± 13.55		4.20 ± 4.15		3.80 ± 3.93		2.33 ± 2.77		4.74 ± 3.88		1.22 ± 1.62	
15	432 (27.2%)	15.43 ± 13.81`		3.91 ± 4.35		3.71 ± 4.09		2.15 ± 2.61		4.57 ± 3.81		1.09 ± 1.64	
> = 16	231 (14.5%)	19.15 ± 14.95		4.54 ± 4.47		4.74 ± 4.60		2.64 ± 2.84		5.81 ± 4.07		1.43 ± 1.47	
Having siblings													
Yes	1,494 (93.9%)	16.61 ± 14.19	0.36	4.19 ± 4.32	0.14	3.98 ± 4.23	0.69	2.28 ± 2.65	0.69	4.93 ± 4.00	0.38	1.23 ± 1.68	0.17
No	97 (6.1%)	15.13 ± 13.23		3.56 ± 3.97		3.67 ± 3.91		2.41 ± 2.86		4.52 ± 3.77		0.98 ± 1.45	
Parents’ marital status													
Original marriage	1,312 (82.5%)	16.68 ± 14.28	0.52	4.18 ± 4.40	0.70	4.00 ± 4.21	0.25	2.31 ± 2.73	0.64	4.95 ± 4.01	0.48	1.24 ± 1.67	0.10
Remarried	152 (9.6%)	14.81 ± 13.05		3.68 ± 3.43		3.39 ± 4.04		2.11 ± 2.36		4.60 ± 4.14		1.03 ± 1.66	
Divorced/Separated	24 (1.5%)	15.63 ± 13.22		4.67 ± 4.29		3.63 ± 4.21		2.46 ± 2.06		4.25 ± 3.67		0.63 ± 1.01	
Widowed	103 (6.5%)	17.26 ± 14.02		4.36 ± 4.12		4.37 ± 4.49		2.23 ± 2.39		5.03 ± 3.56		1.27 ± 1.68	
Family’s economic status													
Very poor	130 (8.2%)	15.75 ± 13.47	0.55	3.90 ± 4.11	0.08	3.62 ± 4.05	0.56	2.16 ± 2.46	0.93	4.95 ± 4.01	0.16	1.12 ± 1.54	0.69
Poor	348 (21.9%)	17.30 ± 14.57		4.47 ± 4.47		4.19 ± 4.32		2.30 ± 2.68		5.13 ± 4.03		1.20 ± 1.73	
Moderate	1,069 (67.2%)	16.45 ± 13.99		4.10 ± 4.23		3.95 ± 4.18		2.30 ± 2.67		4.87 ± 3.97		1.22 ± 1.65	
Wealthy	41 (2.6%)	14.44 ± 16.22		3.85 ± 5.13		3.54 ± 4.46		2.17 ± 3.13		3.61 ± 3.63		1.27 ± 1.90	
Very wealthy	3 (0.2%)	16.67 ± 17.01		0.33 ± 0.58		4.67 ± 7.23		1.67 ± 2.08		7.33 ± 7.02		2.67 ± 2.31	
Living area													
Urban	862 (54.2%)	16.81 ± 14.37	0.47	4.17 ± 4.34	0.88	4.09 ± 4.32	0.37	2.34 ± 2.72	0.58	4.98 ± 3.99	0.39	1.22 ± 1.70	0.86
Rural	729 (45.8%)	16.19 ± 13.86	0.47	4.13 ± 4.25		3.82 ± 4.08		2.23 ± 2.60		4.81 ± 3.99		1.20 ± 1.63	

*p < 0.05.

### 3.2 Correlations between psychological vulnerability, mental health literacy, covariates and adolescent anxiety

Table 2 shows that psychological vulnerability was positively correlated with adolescent anxiety (*r* = 0.48, *p* < 0.01). Furthermore, psychological vulnerability was positively correlated with panic/somatic (*r* = 0.41, *p* < 0.01), generalized anxiety (*r* = 0.49, *p* < 0.01), separation anxiety (*r* = 0.37, *p* < 0.01), social phobia (*r* = 0.38, *p* < 0.01) and school phobia (*r* = 0.37, *p* < 0.01) in adolescents. Among these, the association between psychological vulnerability and generalized anxiety was the strongest. However, no significant correlations were found between the other variables and adolescent anxiety.

**Table 2:** The correlations between psychological vulnerability and anxiety (*N* = 1,591).

Variables	Anxiety *r* (95%CI)	Panic/somatic *r* (95%CI)	Generalized anxiety *r* (95%CI)	Separation anxiety *r* (95%CI)	Social phobia *r* (95%CI)	School phobia *r* (95%CI)
Gender	0.02 (−0.03, 0.08)	0.03 (−0.02, 0.08)	0.02 (−0.03, 0.07)	0.01 (−0.04, 0.06)	0.02 (−0.03, 0.08)	−0.01 (−0.06, 0.04)
Age	0.03 (−0.02, 0.08)	0.01 (−0.04, 0.06)	0.03 (−0.02, 0.08)	0.02 (−0.03, 0.07)	0.04 (−0.01, 0.09)	0.02 (−0.03, 0.08)
Having siblings	−0.02 (−0.07, 0.03)	−0.04 (−0.09, 0.01)	−0.01 (−0.06, 0.04)	0.01 (−0.02, 0.03)	−0.02 (−0.07, 0.03)	−0.03 (−0.08, 0.01)
Parents’ marital status	−0.01 (−0.06, 0.04)	0.01 (−0.03, 0.06)	−0.02 (−0.07, 0.03)	0.02 (−0.03, 0.06)	−0.02 (−0.07, 0.02)	−0.04 (−0.08, 0.01)
Family’s economic status	−0.02 (−0.07, 0.03)	−0.03 (−0.08, 0.02)	−0.02 (−0.06, 0.03)	−0.00 (−0.06, 0.04)	−0.04 (−0.09, −0.02)	0.02 (−0.03, 0.07)
Living area	−0.02 (−0.07, 0.03)	−0.00 (−0.05, 0.05)	−0.02 (−0.07, 0.03)	−0.01 (−0.07, 0.04)	−0.02 (−0.07, 0.03)	0.01 (−0.04, 0.06)
Psychological vulnerability	0.48 (0.44, 0.52)**	0.41 (0.37, 0.46)**	0.49 (0.44, 0.53)**	0.37 (0.33, 0.42)**	0.38 (0.33, 0.42)**	0.37 (0.33, 0.41)**
Mental health literacy	−0.03 (−0.08, 0.03)	−0.01 (−0.06, 0.05)	−0.03 (−0.08, 0.02)	−0.02 (−0.07, 0.03)	−0.03 (−0.08, 0.03)	0.00 (−0.05, 0.05)

**p < 0.01.

### 3.3 The moderating role of mental health literacy

**Figure 1:**
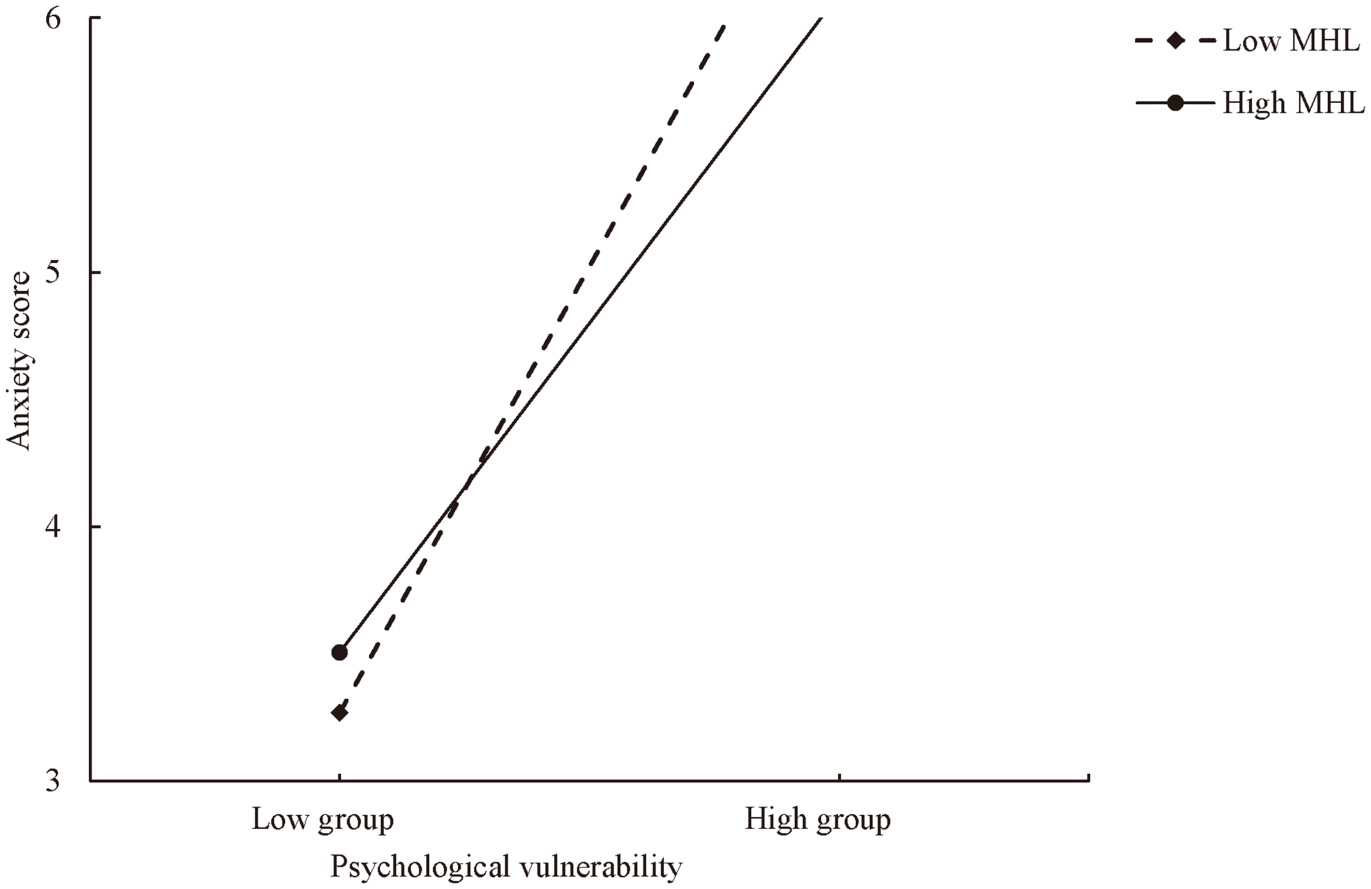
The moderating effect of mental health literacy (MHL) on the relationship between psychological vulnerability and anxiety. The low group is defined as the total score mean of the variable minus one standard deviation, while the high group is defined as the total score mean of the variable plus one standard deviation.

To explore the impact of psychological vulnerability on adolescent anxiety by the moderating effect of mental health literacy, a moderation analysis was performed using Model 1 of PROCESS 3.4. The covariates included gender, age, having siblings, family’s economic status, parents’ marital status, and living area. As shown in Table 3, psychological vulnerability could significantly predict anxiety levels and its dimensions (all *p* < 0.05). The interaction item between psychological vulnerability and mental health literacy in predicting anxiety was marginally significant (*β* = −0.014, *p* = 0.07). Further analysis of the moderating effect on anxiety’s dimensions indicated that mental health literacy significantly moderated the relationship between psychological vulnerability and social phobia (*β* = −0.006, *p* = 0.01). This result suggested that mental health literacy played a significant moderating role in the relationship between psychological vulnerability and adolescent anxiety. To further examine the moderating effect, an interaction effect plot was created for high and low levels of mental health literacy as shown in Figure 1. The line slope in the plot reflects the impact of psychological vulnerability on social phobia. Simple slope tests indicated that mental health literacy significantly weakened the impact of psychological vulnerability on anxiety, showing a significant negative moderating effect. For those with low mental health literacy, as psychological vulnerability increased, adolescent anxiety levels showed a significant upward trend (simple slope = 0.40, *t* = 13.50, *p* < 0.01). For those with high mental health literacy, as psychological vulnerability increased, adolescent anxiety levels also increased significantly (simple slope = 0.29, *t* = 9.98, *p* < 0.01). However, the upward trend in the high mental health literacy group was significantly lower than that in the low mental health literacy group. Therefore, these results suggest that the relationship between psychological vulnerability and anxiety is influenced by the moderating effect of mental health literacy.

**Table 3:** The relationship between psychological vulnerability and adolescent anxiety: the moderating role of mental health Literacy (*N* = 1,591).

Variables	Anxiety		Panic/somatic		Generalized anxiety		Separation anxiety		Social phobia		School phobia	
	*β*	*p*	*β*	*p*	*β*	*p*	*β*	*p*	*β*	*p*	*β*	*p*
Gender	0.63	0.32	0.22	0.27	0.18	0.35	0.10	0.41	0.14	0.44	−0.02	0.82
Age	0.26	0.34	0.02	0.85	0.08	0.34	0.06	0.25	0.10	0.23	0.01	0.81
Having siblings	−0.94	0.47	−0.53	0.21	−0.17	0.66	0.22	0.40	−0.26	0.51	−0.21	0.21
Parents’ marital status	−0.02	0.96	0.33	0.79	0.03	0.77	−0.38	0.63	−0.24	0.83	−0.03	0.57
Family’s economic status	−0.03	0.96	0.08	0.61	0.09	0.55	0.07	0.47	−0.12	0.41	0.07	0.27
Living area	−0.1	0.87	0.09	0.67	−0.14	0.46	−0.05	0.69	−0.00	0.99	0.00	095
Psychological vulnerability	2.6	0.00^**^	0.54	0.00^**^	0.69	0.00^**^	0.41	0.00^**^	0.78	0.00^**^	0.18	0.02^*^
Mental health literacy	0.2	0.15	0.03	0.52	0.04	0.37	0.04	0.21	0.09	0.32	0.01	0.59
Psychological vulnerability × Mental health literacy	−0.01	0.07	−0.00	0.41	−0.00	0.18	−0.00	0.09	−0.01	0.01^*^	−0.00	0.54

*p < 0.05, **p < 0.01.

## 4 Discussion

The psychological vulnerability was positively associated with adolescent anxiety, as well as its five dimensions, including panic/somatic, separation anxiety, social phobia, school phobia, and generalized anxiety. The moderating effect of mental health literacy in the relationship between psychological vulnerability and adolescent anxiety was marginally significant, while it played a significant moderating role in the relationship between psychological vulnerability and social phobia in adolescents. Furthermore, for adolescents with high mental health literacy, as psychological vulnerability increased, their anxiety levels showed a significantly lower upward trend than adolescents with low mental health literacy.

Psychological vulnerability was positively associated with adolescent anxiety in line with previous studies (Lockhard et al., 2001; Gallagher et al., 2014; Chorpita and Barlow, 2018). Adolescent psychological vulnerability might stem from a lack of control and autonomy in their early childhood environments, leading to the development of negative and self-doubting cognitive styles (Chorpita and Barlow, 2018). These negative cognitive styles, once established, have the potential to link with anxiety symptoms (Keefer et al., 2018). The psychological vulnerability of adolescents is often intertwined with childhood adversities, which can serve as a potential marker of dysregulated stress responses, consequently heightening the risk of adolescents developing anxiety symptoms (Chen et al., 2021). The Triple Vulnerability Theory posits that adolescent anxiety may arise from an interacting set of three vulnerabilities or diatheses, including generalized and heritable biological vulnerability, generalized psychological vulnerability, and specific psychological vulnerability related to certain life circumstances (Suárez et al., 2009). More critically, adverse childhood experiences are strongly correlated with an increased risk of suicidal behaviors (Baldini et al., 2023). Research has indicated that anxiety is linked to suicide, which poses a significant threat to adolescents’ health and well-being, and that psychological vulnerability may exacerbate this association (Conner et al., 2001; Baldini et al., 2024).

The psychological vulnerability was positively associated with panic/somatic, separation anxiety, social phobia, school phobia, and generalized anxiety. Higher levels of psychological vulnerability are associated with an exaggeration of bodily sensations, which serves as an interpretation of a threat, increasing the likelihood of adolescents experiencing panic or somatic symptoms (Eley et al., 2007), in line with the significant correlation between psychological vulnerability and panic/somatic. Experiencing adverse events, which contribute to the formation of an individual’s cognitive vulnerability, can increase the risk of developing generalized anxiety disorder (Gosselin and Laberge, 2003; Chambers et al., 2004). Therefore, generalized anxiety is considered to be the outcome of psychological vulnerability triggered by the tension resulting from negative events (Gosselin and Laberge, 2003), affirming the correlation between psychological vulnerability and generalized anxiety. The psychological vulnerability in adolescents primarily originates from familial control and conflict, leading to an insecure-ambivalent parent–child attachment which is related to anxiety symptoms in early adolescence (Warren et al., 1997; Cowan et al., 1996; Cassidy and Berlin, 1994; Eisen and Schaefer, 2007), explaining the association with separation anxiety. The psychological vulnerability related to social phobia can be manifested as heightened vigilance in the face of social threat cues (Hope et al., 1990). This is because individuals with high psychological vulnerability tend to focus on negative social cues and then internalize them (Hope et al., 1989). Hence, they are more susceptible to self-criticism (Hope et al., 1989). The level of self-criticism is significantly correlated with the severity of social anxiety symptoms (Cox et al., 2002). School phobia is linked to developmental psychological vulnerability factors (Ek and Eriksson, 2013). Excessive psychological vulnerability to negative academic performance can trigger anxiety in adolescents, ultimately increasing the risk of school avoidance (Ek and Eriksson, 2013), which explains the association between psychological vulnerability and school phobia.

The moderating role of mental health literacy (MHL) in the relationship between psychological vulnerability and adolescent anxiety is worth noting, as the overall interaction approached statistical significance (*p* = 0.07), suggesting a trend-level buffering effect. Although not reaching conventional levels of significance, this marginal result indicates that MHL may still serve as a potential protective factor worthy of further exploration in future longitudinal or larger-sample studies. A high level of mental health literacy is beneficial for adolescents to recognize anxiety symptoms and proactively seek professional help (Morgado et al., 2021; Coles and Coleman, 2010; Coles et al., 2016), consequently promoting early intervention and enhancing long-term outcomes (Clarke et al., 2006). On the other hand, enhanced mental health literacy empowers adolescents with better self-regulation skills for their emotions when they know how to prevent mental disorders (Nogueira et al., 2023; Jorm, 2012), which helps them reduce psychological vulnerability (Brooks et al., 2022; Beauregard, 2007; Nelson et al., 2010). Therefore, a high level of mental health literacy weakens the relationship between psychological vulnerability and adolescent anxiety.

Interestingly, MHL significantly moderated only the relationship between psychological vulnerability and social phobia, but not other dimensions of anxiety, such as generalized or panic-related symptoms. One possible explanation lies in the distinct interpersonal nature of social phobia, which is more directly influenced by adolescents’ understanding of social functioning and their awareness of emotional difficulties in social contexts. Given that social-personal difficulties can significantly contribute to social phobia and suicidality among adolescents among adolescents with mental health disorders (Baldini et al., 2025; Mallya et al., 2015), improving MHL may play a pivotal role in enhancing interpersonal skills, mitigating social anxiety, and attenuating the impact of psychological vulnerability specifically in this domain.

The cultural context in China may further explain the prominence of social phobia. Deep-rooted Confucian values place strong emphasis on academic achievement and social harmony. Chinese adolescents often face immense academic pressure and limited opportunities for developing social competencies, as schools prioritize examination performance over interpersonal development (Zhuang et al., 2025). As a result, social anxiety has emerged as one of the most prevalent and recognizable forms of adolescent anxiety. Previous studies have shown that social phobia is more readily recognized than other anxiety disorders among Chinese youth (Hadjimina and Furnham, 2017). In collectivist cultures like China, where interpersonal harmony and social evaluation are highly emphasized, adolescents are particularly sensitive to social approval (Gureje et al., 2006; Ng, 1997). Consequently, those with low MHL may misinterpret social phobia symptoms as mere shyness or personality flaws, reducing their likelihood of seeking help (Jorm et al., 2007). In China, mental health knowledge among Chinese adolescents mainly derives from formal education in school, which bears a certain Chinese characteristic (Gong and Furnham, 2014). It is plausible that education plays a more prominent role in shaping adolescents’ understanding of social anxiety specifically. Thus, enhancing school-based MHL education may be particularly effective in mitigating the impact of psychological vulnerability on social phobia in the Chinese context.

A notable strength of this study lies in its concurrent examination of both intrinsic risk and protective factors originating from adolescents themselves about adolescent anxiety, especially social phobia. It is a reminder for clinical psychologists that high psychological vulnerability serves as a susceptible marker for adolescent anxiety. In contrast, mental health literacy is a modifiable trait factor that can lower the risk of anxiety among adolescents. However, nowadays most adolescents are lacking in sufficient mental health literacy, making it necessary and urgent to promote mental health knowledge among the general population (Brooks et al., 2022). In this situation, public health interventions should be widely used to reduce treatment barriers and provide social support (Brooks et al., 2022). Adequate social support can not only reduce the stigmatization and marginalization of adolescents with mental illnesses in society (Brooks et al., 2022) but also contribute to lowering the risk of adolescent anxiety (Qi et al., 2020). This underscores the importance for educators and teachers to emphasize the dissemination of accurate and professional mental health knowledge during a psychology class. Efforts should be made to develop educational resources and public awareness programs aimed at cultivating mental health literacy among adolescents. Future research should explore how these results manifest in societies with different cultural values, such as those emphasizing individualism over collectivism, considering variations in educational systems.

This study has several limitations that should be acknowledged. First, it employed a cross-sectional design, which precludes the establishment of causal relationships between psychological vulnerability and adolescent anxiety. As such, causal inferences cannot be made. Future research using longitudinal or prospective cohort designs is needed to clarify the directionality and underlying mechanisms of these associations, particularly in accounting for autoregressive effects and time-varying moderating influences (Zhuang et al., 2025). Second, the sample was drawn exclusively from a single province in China, which may limit the generalizability of the findings. Cultural, educational, and regional differences may affect the observed relationships, and caution should be exercised when extending these results to adolescents in other provinces or international settings. Replication in more diverse and representative samples is warranted. Third, the study did not include biological or genetic variables, which are important factors in understanding the etiology of adolescent anxiety and deserve further exploration in future research.

## 5 Conclusion

The results of this study showed that psychological vulnerability was positively associated with adolescent anxiety, including panic/somatic, separation anxiety, social phobia, school phobia, and generalized anxiety, and that mental health literacy weakened the relationship between psychological vulnerability and social phobia. These findings call on parents to pay attention to negative life events in children, aiming to minimize psychological vulnerability. Clinical psychologists can predict anxiety symptoms in adolescents through the psychological vulnerability traits they observe. Besides, they could potentially prevent or alleviate social phobia among adolescents by promoting mental health knowledge. Educators and teachers should put in more effort and explore various approaches to cultivate mental health literacy among adolescents.

## Data Availability

The raw data supporting the conclusions of this article will be made available by the authors, without undue reservation.
